# SEM-EDS investigation on PM10 data collected in Central Italy: Principal Component Analysis and Hierarchical Cluster Analysis

**DOI:** 10.1186/1752-153X-6-S2-S3

**Published:** 2012-05-02

**Authors:** Alessandra Genga, Federico Baglivi, Maria Siciliano, Tiziana Siciliano, Marco Tepore, Gioacchino Micocci, Carmela Tortorella, Domenico Aiello

**Affiliations:** 1 Department of Materials Science, University of Salento, via per Arnesano, 73100 Lecce, Italy; 2 Enel - Engineering and Innovation - Technical Research Area - Litoranea S.na Brindisi Casalabate - Cerano - Tuturano 72020 Tuturano (Br), Italy

## Abstract

**Background:**

Principal Component Analysis (PCA) and Hierarchical Cluster Analysis (HCA) were applied on PM10 particle data in order to: identify particle clusters that can be differentiated on the bases of their chemical composition and morphology, investigate the relationship among the chemical and morphological parameters and evaluate differences among the sampling sites. PM10 was collected in 3 different sites in central Italy characterized by different conditions: yard, urban and rural sites. The concentration of 20 chemical parameters (C, O, Na, Mg, Al, Si, P, Cd, Cl, K, Ca, Sn, Ti, Cr, Mn, Fe, Co, Ni, Cu, Zn) were determined by Scanning Electron Microscopy – Energy Dispersive X-ray Spectroscopy (SEM-EDS) and the particle images were processed by an image analysis software in order to measure: Area, Aspect Ratio, Roundness, Fractal Dimension, Box Width, Box Height and Perimeter.

**Result:**

Results revealed the presence of different clusters of particles, differentiated on the bases of chemical composition and morphological parameters (aluminosilicates, calcium particles, biological particles, soot, cenosphere, sodium chloride, sulphates, metallic particles, iron spherical particles). Aluminosilicates and Calcium particles of rural and urban sites showed a similar nature due to a mainly natural origin, while those of the yard site showed a more heterogeneous composition mainly related to human activity. Biological particles and soot can be differentiated on the bases of the higher loads of Fractal Dimension, which characterizes soot, and content of Na, Mg, Ca, Cl and K which characterize the biological ones. The soot of the urban site showed higher loadings of Roundness and Fractal Dimension than the soot belonging to the yard and rural sites, this was due to the different life time of the particles. The metal particles, characterized mainly by the higher loading of iron, were present in two morphological forms: spherical and angular particles. The first were generated by a fusion process at high temperature, while the second one had crustal origin (those characterized by typical terrigenous elements) and also human origin.

**Conclusion:**

In this work a protocol for the morphological-chemical characterization of single particles has been developed. SEM analysis allows to classify particles in 10 different families and PCA and HCA have provided information about the sources of PM and similarities and differences among the sites.

## Background

Particulate air pollution is one of the most pressing problems of cities in developed countries. The undeniable effect is the increasing number of people affected by problems of respiratory and cardiovascular health [1-3].

The physical-chemical, morphological and dimensional determinations of the complex mixture of organic and inorganic particulates are one of the major aspects for their characterization and for identification of emission sources that contribute to particulate air concentrations [4]. In fact particles have different shapes, sizes and chemical composition in relation to emission sources [5-8]. These characteristics are certainly influenced by the residence time of particles in the atmosphere and by the meteorological condition, but basically they retain some memory of emissive sources in the atmosphere. These parameters are therefore crucial to identify possible emission sources [9] and assess the well known health and environmental effects [10-12].

In particular the size and especially the shape of the particles appear to be the decisive parameter for the assessment of risks to health. As documented, there is a correlation with the pathogenic action, not with the chemical composition of particles or fibers breathed, but even more with the form of these [13-17].

Scanning electron microscopy analysis is a useful tool, which allows the characterization of individual particles within an aerosol overcoming the limitations imposed by bulk chemical analysis [18-23]. The morphology and composition of individual particles may constrain aerosol sources and provide insight into the transport mechanisms that have influenced the particles [24,25].

In particular, some documented studies have been concerned to relate the composition and morphology of particulate highlighting the special relationship with atmospherical conditions [26,27].

In this work a protocol for a systematic SEM study of filters belonging to three sites was carried out. The three sampling sites for atmospheric monitoring have been located along costal areas in central Italy. The first site was selected within a thermal power station which was under construction in the sampling period. The second sampling site was within a city park in an urban area, while the third site was near a rural area, north of the power station. The three sites are about 5 Km distant from each other. The collected samples represent sites characterized by different conditions: a yard site, an urban site and a rural site.

The urban center has a population of over 50,000 inhabitants. The area is predominantly agricultural, with particular abundance of arable land, and it presents shrub vegetation and agricultural areas integrated with natural areas. The town has industrial settlements occupying 6% of the municipal area, the urbanized areas are concentrated mainly on the coastline, while the inland presents lower levels of urbanization. Moreover, during the sampling period, there was a high density of vehicular traffic with an average of 10,000 vehicles per day and 700 trucks along the Motorways of the Sea, due to the presence of harbors.

In this study some interesting observations can be inferred on the different shapes and chemical composition of the individual particles on the filters. Multivariate statistical analysis was applied on the particles morphological-chemical parameters that were analyzed and measured by SEM-EDS and Image Pro Analyzer software. Principal Component Analysis (PCA) and Hierarchical Cluster Analysis (CA) allow us to investigate the correlations among the identified variables and the set of particles, to identify clusters of particles that can be differentiated on the bases of their chemical composition and morphology and to evaluate the differences among the sampling sites. In this study the application of statistical methods on a data set, determined by individual particle analysis with the SEM technique, allows us to obtain information related to morphological and chemical properties that can not be derived from an analysis of the filter through total destructive techniques like Graphite Furnace - Atomic Absorption Spectrometry (GF-AAS), Inductive Coupled Plasma – Optical Emission Spectroscopy (ICP-OES) or other methods.

## Results and discussion

### SEM analysis

Examining SEM images and the spectrum of the single particles these were grouped and labeled as: particles of aluminosilicates (AlSi), particles rich in calcium (Ca), carbonaceous particles of biological origin (Cb), soot particles (carbon particles from incomplete combustion of organic matter) (Cs), cenosphere (Cc), salts of sodium chloride (Cl), sulfates (Sc), metal particles (Me), spherical particles of iron (Fe), various (particles without a characteristic spectra) (Va). These particles were characterized as follow:

*Aluminosilicates* (45.1% of particles): these particles are composed primarily of Si, Al, Ca or Si, Al, K (e.g. feldspar) and Si, Al or Si, Al, Fe (e.g. clay), their origin is mainly crustal, but they can also come from the erosion of building products and road dust. Other elements are present in minor concentration in the aluminosilicate particles and they are Na, Mg, Ti, Mn, Ni and Zn. Within the group of aluminosilicates some crustal particles rich in Si (principally quartz) are included, for a similar origin. These particles mainly present an angular shape, ranging from polyhedral to a sharp one.

*Calcium-rich particles* (21.7% of particles): particles with a high concentration of calcium are included in this group. They are calcium carbonate, calcium oxide and particles with minor concentration of elements such as Al, K and Si. These particles can be related to crustal source and they can belong to yard activities such as lime production and the use of cement.

These particles are present in the whole studied size range and they show a greater Roundness than the aluminosilicate particles.

*Biological carbon* (4.8% of particles): biological particles (pollen, spores and plant fragments) show an high content of carbon and oxygen and sometimes they are characterized by minor content of elements such as Na, Mg, P, K and Ca. They are characterized by regular and symmetrical shapes, ranging from spherical to elliptical shapes.

*Soot* (7.2% of particles): these particles are an agglomeration of carbon particles resulting from the incomplete combustion of organic matter, they are characterized by 80% - 96% of carbon in relationship to the physical-chemical condition of combustion. They originate from the combustion of burning oil, gasoline, diesel, fuel oil, paraffin, butane; they are, therefore, an important tracer of vehicular traffic.

The structure of soot particles are strictly related to their age, in fact their shape range from a linear to a more and more branched structure, where the first one is related to a newly formed soot and the second one to an advanced agglomeration. Furthermore this type of particle is characterized by an higher value of Fractal Dimension (average value of 1,16 + 0,07) than the other particles families (AlSi 1.10+0.04, Ca 1.08 + 0.03, Cb 1.08 + 0.04, Cc 1.08 + 0.04, Cl 1.07 + 0.03, Fe 1.06 + 0.01, Me 1.07 + 0.02, Sc 1.10 + 0.04).

*Cenosphere* (0.7% of particles): these particles are carbonaceous and they originate from incomplete combustion processes of diesel and fuel oil. These particles present a minor amount of S and metals such as V, Ni, Fe, Ti. They are characterized by a spherical shape.

*Salts of sodium chloride* (3.5% of particles): these particles are essentially composed of Na and Cl, sometimes traces of Mg, K, Ca and S are detected. NaCl particles are mainly due to marine aerosols.

*Calcium sulfate* (1.7% of particles): these particles are originated by acid-base neutralization reactions in the atmosphere and from the deterioration of building surfaces (e.g. the reaction of marble and limestone with sulfur compounds in the atmosphere). Calcium sulfate is also used for the production of cement and it is a secondary product of desulphurization of flue gas. The shape of these particles is typically symmetrical and elongated, even if there are irregular examples of particles, too.

*Metal* (7.5% of particles): these are particles with a high metal content such as Fe, Zn, Ti, Cu, Mn and Cr. They originate from different sources depending on the metal content: particles containing mainly Fe, for example, can be of crustal origin, but may also come from human activities such as industrial processes, abrasion of metallic materials and traffic-related sources.

*Spherical particles of iron* (0.4% of particles): These particles consist exclusively of iron oxide. Their size ranges between 0.2 and 2 μm and they present a peculiar morphology, in fact they are characterized by a perfect sphericity indicating their smelting iron origin or metallurgical activities in general. They are characterized by shape factor (Roundness) of 0.9 + 0.13 and Fractal Dimension of 1.06 + 0.01.

*Various* (6,9% of particles): these are particles without a characteristic spectra with the presence of low analytic signals. Moreover, it has to be pointed out that these particles are the shortest (diameter near to 0.6 μm). The absence of characteristic spectra for these kinds of particles is mainly due to an instrumental limit of SEM – EDS as it will be discussed in the method section for particles with a diameter less than 0.6 μm. Particles classified as 'various' are characterized by low weight percentages of the investigated elements and their classification turns out to be difficult.

### **Statistical analysis**

The Principal Component Analysis was performed on the entire data set and on different sub-data sets in order to study the correlation among the variables and the particles in each sampling site, to obtain information on the emissive pollution sources and to investigate the differences among the three sampling sites. Co, Ti and P were not considered because they are detected only in few particles while Sn and Cd were excluded by the analyses because of spectral interferences probably due to signs of K and Ca.

**Figure 1:**
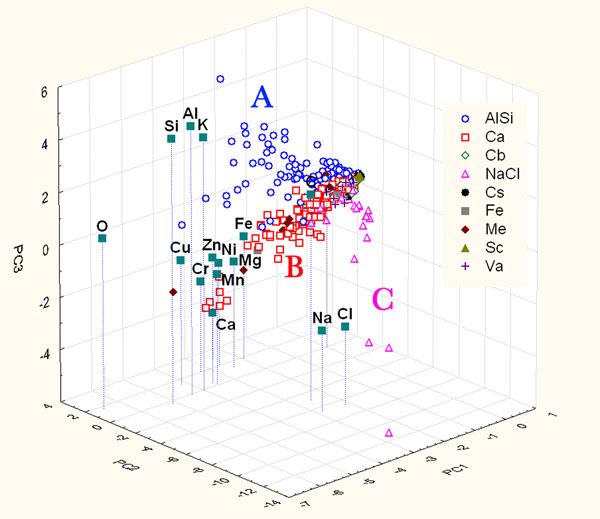
Score and loading plot of the first three PCs considering the only contribution of chemical parameters of particles sampled on Yard site. A, B and C represent clustering group.

The PCA of the chemical data of the particles belonging to the yard site was performed using the following chemical variables: C, O, Na, Mg, Al, Si, Cl, K, Ca, Cr, Mn, Fe, Ni, Cu, and Zn. These variables have provided a set of structured data in a multidimensional matrix (15 variables for 494 samples). Figure 1 shows the contributions to the first three principal components to represent the distribution of particles. The first three principal components can explain 46% of the total variance.

The score analysis shows the presence of particle clusters which overlap at the zero values of the PCs, this is due to the evidence that particles near the zero values are characterized by a very low dimension and thus by low signals in the EDS spectra.

Examining Figure 1, the high correlations among Na-Cl, Al-Si-K-O and Ca with Fe-Cr-Ni-Mg-Zn-Mn-Cu are evident. These correlations are an index of the same sources for the correlated elements: the first ones may originate from sea spray which is characterized by the presence of NaCl, the second ones could be due to crustal source mainly composed of Al, Si. The third source, characterized by calcium and other metal elements, could be an anthropic source for example a PM emission traffic related one (gasoline/diesel emission and wear particles generated from brake materials) [28] and yard activities that lead to the presence of Ca compounds (coal and so on).

Loading analysis explains that the discrimination among cluster along PC2, leading to the separation of A and B clusters from C, is mainly due to the contribution of Na-Cl, while along PC3, leading to the separation of A cluster from B, mainly due to the contribution of Al-Si-K.

This evidence allows us to assume that different clusters define different particle families: aluminosilicates (Cluster A), chloride (Cluster C) and rich in calcium and metals particles (Cluster B). Moreover, other interesting information can be drawn analyzing the arrangement of particles in the clusters. In particular the particles characterized by the highest loadings of the parameters are morphologically the biggest ones of the analyzed set.

**Figure 2:**
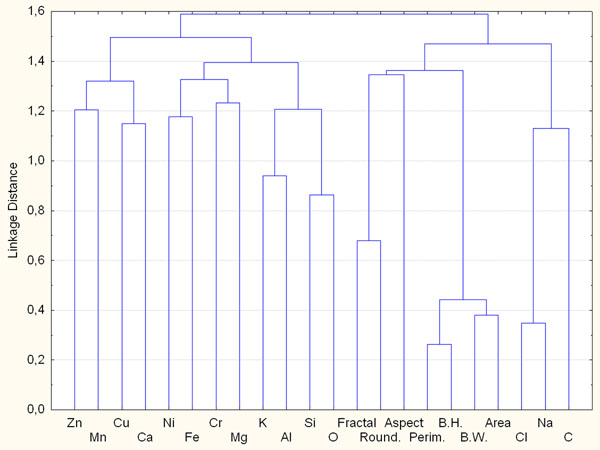
Hierarchical clustering dendrogram obtained with Ward’s method considering the only contribution of chemical parameters of particles sampled on Yard site.

The hierarchical cluster analysis (Figure 2) confirms the correlations among the different variables observed in the PCA: Na-Cl, Al-Si-K-O linked to elements Fe-Cr-Ni-Mg, and Zn-Mn-Ca-Cu. The link among group Al-Si-K-O and Fe-Cr-Ni-Mg is partly due to the presence of terrigenous particles with a low amount of Fe and Mg (e.g. (Ca_2_Mg_5_)Si_8_O_22_(OH)_2_ (amphibole), (Mg,Fe)_2_SiO_4_ (Olivine), (Mg, Fe)_2_SiO_3_ (pyroxene) and K(Mg, Fe)_3_Si_3_O_10_(OH)_2_ (biotite)), and partly due to the presence of road dust characterized by aluminosilicates with the presence of metals, this is an index of vehicular traffic and industrial activity. In general a wide compositional range of crustal particles has been observed in this site, this is manly due to the contribution of building material and soil moving that characterize the yard activity.

**Figure 3:**
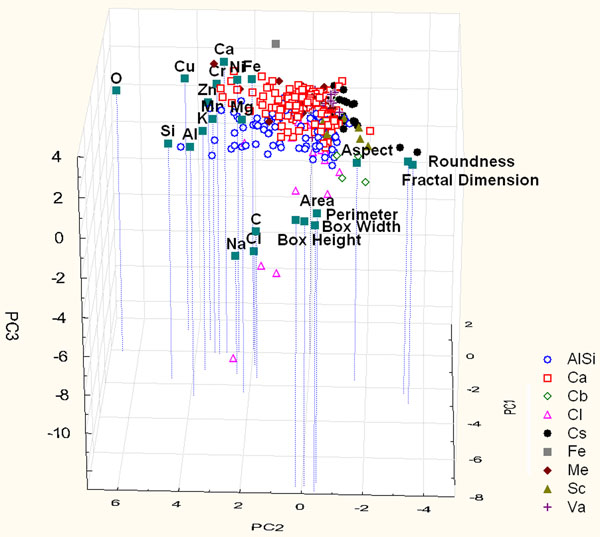
Score and loading plot of the first three PCs considering the contribution of chemical and morphological parameters of particles sampled on Yard site.

The role of morphological variables has also been studied adding to the chemical data set by the morphological data. The score and loading plots (Figure 3) of the first three PCs explain 43% of the total variance. This plot doesn’t show an evident differentiation among the particles, while it is possible to notice evident correlations among the morphological variables (Area - Box Width - Box Height - Perimeter and Fractal Dimension - Roundness) and among the chemical variables (Na-Cl and all the other chemical elements). In fact PC2 and PC1 separate morphological parameters from chemical ones and they contribute to separate the morphological variables in the two different clusters highlighted before. Furthermore PC3 separate Na-Cl from the remaining chemical variables.

Figure 3 shows that soot particles are characterized by high loading of shape variables (Fractal Dimension e Roundness), while biological one are influenced principally by high loading of dimensional variables (Area - Box Width – Box Height - Perimeter). These results highlight the importance of morphological variables for soot and biological particle discrimination, in fact these particles are not differentiated by the use of only chemical variables in the PCA (Figure 1). The dispersion of the other particles is due to the combined influence of chemical and dimensional variables.

The hierarchical cluster analysis shows the same correlations identified using only chemical variables (Al-Si-K-O, Na-Cl, Zn-Cu-Mn-Ca, Fe-Cr-Ni-Mg) and correlations among morphological parameters: in particular dimensional variables (Area, Perimeter, Box Width and Box Height) and shape variables (Roundness and Fractal Dimension) are closely correlated.

**Figure 4:**
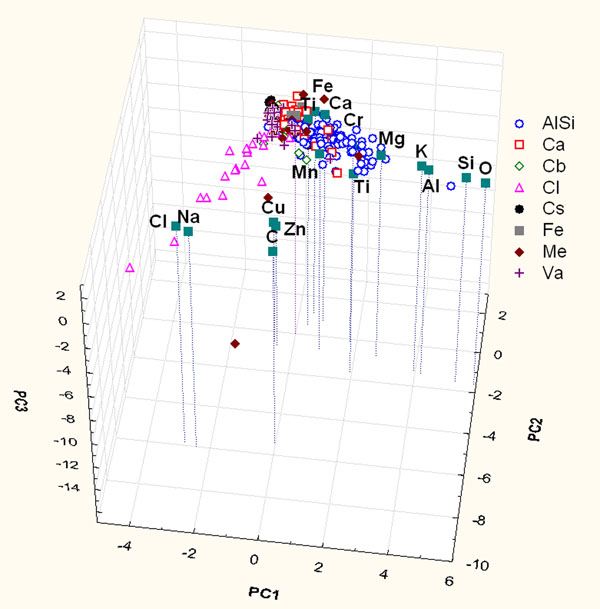
Score and loading plot of the first three PCs considering the only contribution of chemical parameters of particles sampled on rural site.

PCA and HCA of the compositional data matrix of the rural site (variables C, O, Na, Mg, Al, Si, Cl, K, Ca, Cr, Mn, Fe, Ni, Cu, and Zn for 317 samples) were performed. Figure 4 shows the contributions to the first three principal components (43% of the total variance) to represent the distribution of particles.

Examining Figure 4 it is evident that also in this plot the particle clusters overlap at the zero values of the PCs for the same reason as the precedent analysis. It is possible to notice that PC1 is characterized by high loadings of Al-Si-K-O; PC2 is characterized by high loadings of Na and Cl and PC3 is characterized by high loadings of Cu and Zn. These associations suggest common sources for the correlated elements and of the presence of particles deriving from: crustal sources (minerals containing aluminosilicates mentioned in the precedent paragraph), sea spray and the last one can be ascribable to anthropic sources like brass, copper and zinc alloys and traffic related compounds. It has to be pointed out that in this figure only NaCl and two Cu-Zn particles are differentiated from the other particles; the aluminosilicate particles are characterized by higher loading of Al-Si-K-O and that the carbonaceous particles (both biologic and anthropic one), in the same way as Figure 1, remain near the zero value of the PCs.

**Figure 5:**
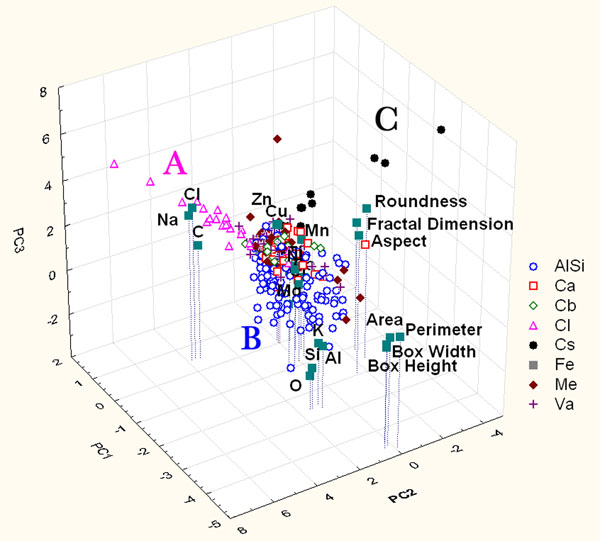
Score and loading plot of the first three PCs considering the contribution of chemical and morphological parameters of particles sampled on rural site. A, B and C represent clustering group.

Then the role played by the parameters related to the morphology has been investigated. The score and the loading plot of the first three PCs are shown in Figure 5. It is possible to note evident correlations among the morphological variables (Area with the associated Box Width - Box Height – Perimeter and Fractal Dimension with the associated Roundness) on one hand and among the chemical variables (Na-Cl, Al-Si-K-O, Zn-Cu and among all the remaining chemical elements) on the other hand. These clusterings determine the separation of cluster A (NaCl particles) from Cluster B (mainly composed of aluminosilicate particles) and then the separation of Cluster C (Soot particles). This last cluster can be differentiated now because it is mainly characterized by high loadings of shape variables (Fractal Dimension and Roundness).

Moreover other interesting information can be drawn analyzing the arrangement of particles in cluster B. In particular this cluster shows a high dispersion of scores and the particles inside it are differentiable in accordance with their dimension (Area, Box Width, Box Height and Perimeter). These parameters are enough to determine intra-cluster discrimination without modifying inter-cluster variance. Even if also in this figure the carbonaceous biological particles are not differentiated in a single cluster and they are in the cluster with the aluminosilicates, they seam to be partially influenced by the dimension parameters. These results highlight again the importance of morphological variables for carbonaceous particles discrimination and in particular for soot particles.

All these inferences are reinforced by the results of hierarchical cluster analysis which confirms the presence of: crustal sources composed mainly of Al-Si-K-O and/or rich in Ca (Ca-Mg-Cr compounds), sea spray characterized by NaCl and anthropic sources characterized by the correlation Zn-Cu then associated to aluminosilicates (Al-Si-K) (traffic related sources or anthropic sources in general) or metallic particles characterized by Ni-Mn-Ti. Moreover the correlation among Fractal Dimension and Roundness are suggestive of the presence of soot, as said before.

**Figure 6:**
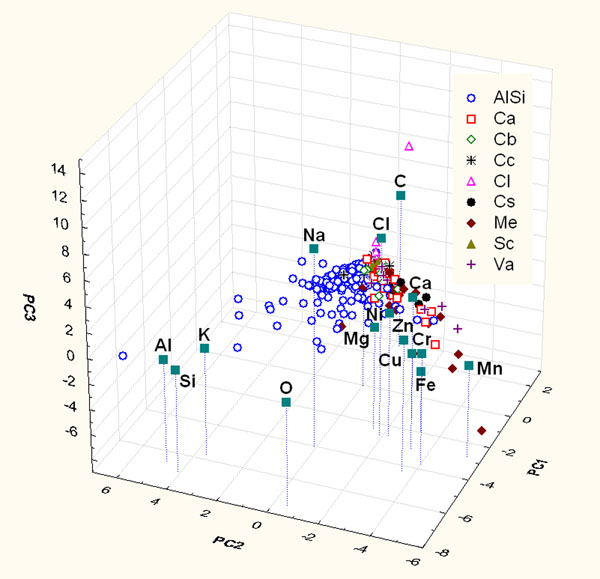
Score and loading plot of the first three PCs considering the only contribution of chemical parameters of particles sampled on urban site.

The PCA of the urban site was performed using chemical variables, specifically the elements C, O, Na, Mg, Al, Si, Cl, K, Ca, Cr, Mn, Fe, Ni, Cu, and Zn. These parameters have provided a set of structured data in a multidimensional matrix (15 variables for 524 samples). Figure 6 shows the contributions from the first three principal components to represent the distribution of particles. The first three principal components can explain 45% of the total variance.

Examining Figure 6, the high correlations Na-Cl, Al-Si-K and Ca associated to Fe-Cr-Ni-Mg-Zn-Mn-Cu are evident. The correlations, index of the same sources for the correlated elements, allow us to identify different clusters of parameters: the first one is due to NaCl particles, the second one to aluminosilicates and the last one to the compounds with calcium and other metal elements.

Even in this site there correlations are due to the presence of sea spray, crustal sources and anthropic sources (mainly traffic related). The Hierarchical Cluster Analysis shows correlations among different variables that confirm the score and loading analysis: Al-Si-K-O, Na-Cl, Cu-Fe-Mn-Ca-Mg, Ni-Zn-Cr.

Moreover it has to be pointed out that the particles of the urban site are not differentiated in well defined clusters, even if an intra-cluster differentiation can be seen, in fact the particles can be grouped in accordance to their chemical composition. This evidence can be due to the contamination of natural sources with anthropic elements.

It is important to note that urban and rural sites are characterized by a smaller amount of particles rich in calcium than the Yard site, and also by a lower dispersion of silicate scores. These results are due to the low heterogeneity of silicates in the first two sites: silicates found here are mainly characterized by crustal local origin; while in the yard site silicate presence is also due to the presence of building material chemically different from the local crustal materials. Also Ca particles are mainly due to building activity; these hypothesis are reinforced by HCA results which show a strong correlation between Si-Al then associated to K, in urban and rural sites (Si, Al and K are characteristic of the local terrigenous minerals), while in the yard site two linked sub-groups of correlated parameter are evident (Si-O and K-Al). This evidence can be due both to Al-Si-K compounds and to a large presence of SiO_2._ The urban site is also characterized by a lower amount of NaCl than the other sites.

**Figure 7:**
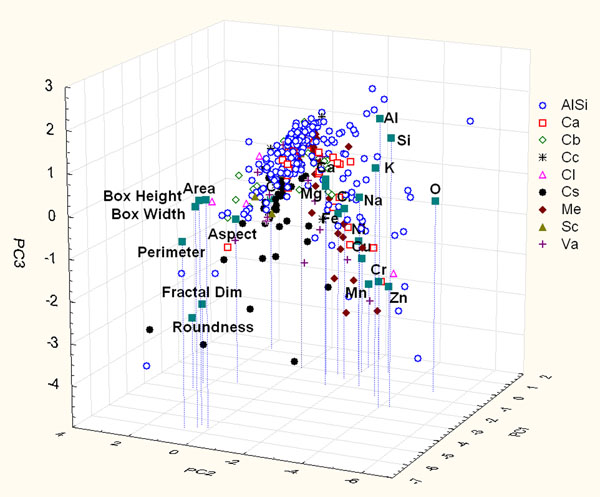
Score and loading plot of the first three PCs considering the contribution of chemical and morphological parameters of particles sampled on urban site.

When morphological parameters are considered too (Figure 7), the PCA on the urban data set (the first three PCs are able to explain 43.8% of total variance) shows five distinct parameter clusters: Al-Si-K, Na-Mg-Cl-Fe-Ca, Ni-Cr-Cu-Zn-Mn and the morphological parameters: Area – Perimeter - Box Width - Box Height and Roundness - Fractal Dimension. It is possible to notice that PC1 separates morphological dimensional parameters (Area - Perimeter - Box Width - Box Height) from chemical ones, and PC2 separates shape parameters (Fractal Dimension and Roundness) from chemicals ones. PC3 separates Al-Si-K from metals such as Cr, Mn, Cu and Zn and dimensional parameters from shape parameters.

Examining Figure 7, it is evident that most of AlSi particles are characterized by high loadings of morphological dimensional parameters while smaller amounts are characterized by chemical parameters along PC2. This evidence can be justified considering that the larger particles of this site are the aluminosilicate ones, while the other particles are mainly characterized by lower dimensions.

It is also important to note that the carbonaceous particles, in particularly those classified in the soot family (Cs), are discriminated along the PC2 and PC3, by higher loading of morphological shape parameters (Roundness and Fractal Dimension). This result confirms the importance of the morphological variables for soot discrimination. It has to be pointed out that soot aggregate particles in the urban site are more abundant than those in the rural and yard site, which suggests that the pollution caused by vehicle exhaust in this site is more serious than in the other two areas. This is reasonable because the traffic is busier in the urban area than in the rural area and it is in accordance with the general anthropic metal contamination of the urban particles. Moreover, soot of the yard and the rural sites shows Roundness values and Fractal Dimension values greater than the soot found in the urban site; this difference can be explained considering the age of particles: young particles are less compact and they are characterized by branches that raise Roundness and Fractal Dimension values; older particles tend to reach Roundness values close to one and the Fractal Dimension decreases, it is due to the decreasing of isolated branches of the particles. The soot particles, which are found in the urban site, have dimension from few nm to 10 μm of diameter, this evidence is due to the presence of both soot of new formation (the finest one) and an older soot of greater dimension. This situation is reasonable with a high traffic area and it confirms the higher contribution of anthropic sources to the PM composition of this site. The other two sites, instead, are characterized by soot of new formation and it is due to the minor amount of traffic density.

The Hierarchical Cluster Analysis confirms the correlations observed in the PCA.

The statistical analysis has been performed also on complete dataset composed of all the particles (1334) of the three sites.

**Figure 8:**
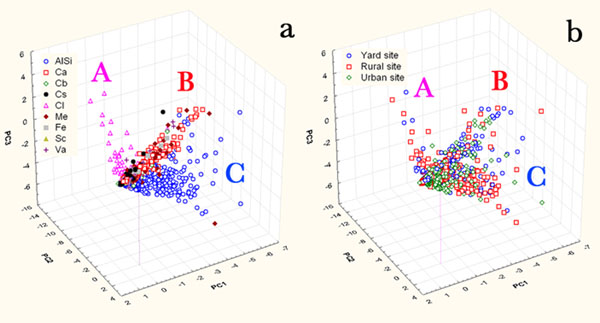
Score plot of the first three PCs considering the contribution of chemical parameters of particles sampled on the three investigated sites (a – particles differentiated by composition, b – differentiated by site of sampling). A, B and C represent clustering group.

**Figure 9:**
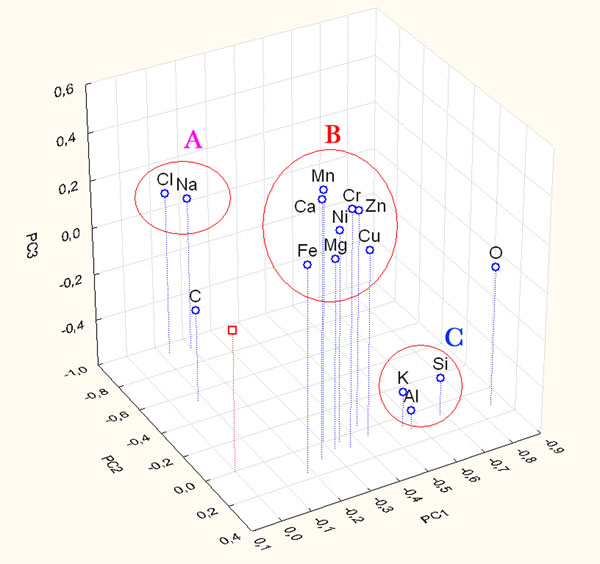
Loading plot of the first three PCs considering the contribution of chemical parameters of particles sampled on the three investigated sites. A, B and C represent clustering group.

Considering the chemical parameters (C, O, Na, Mg, Al, Si, Cl, K , Ca, Cr, Mn, Fe, Ni, Cu, and Zn), the first three PCs (Figure 8a), which explain the 43% of the total variance, have clustered the particles in three clusters in accordance to their chemical composition (Figure 9) and not to their sampling sites (Figure 8b); in fact cluster A, characterized by high loadings of Na and Cl, is composed of NaCl particles; cluster B by Ca and other metal elements and a more abundant and dispersed cluster C characterized by Al-Si-K-O. Figures 9 and 8 confirm the previous analysis: NaCl are mainly present in the rural and yard site; Ca particles in the yard site and the soot in the urban site.

**Figure 10:**
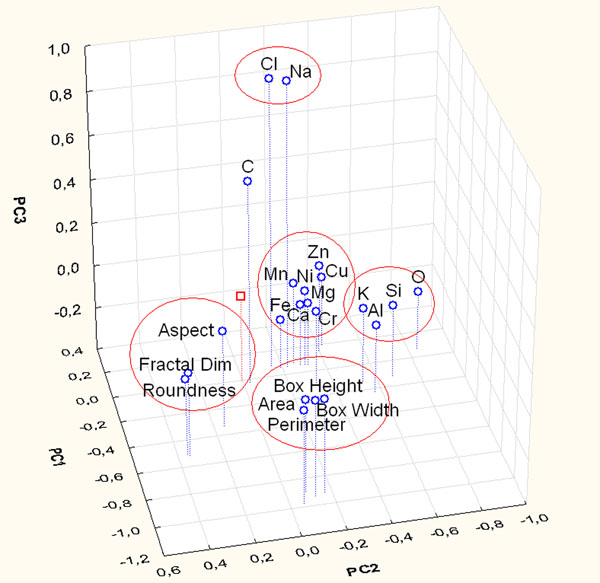
Score and loading plot of the first three PCs considering the contribution of chemical and morphological parameters of particles sampled on the three investigated sites.

**Figure 11:**
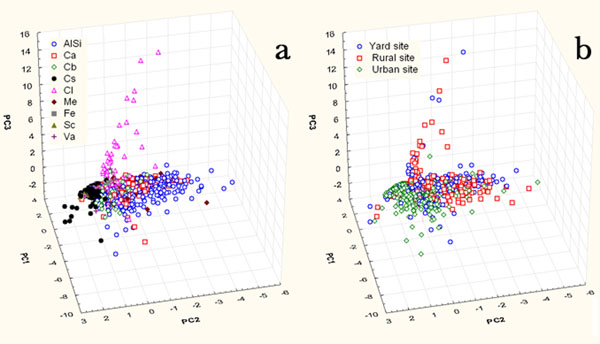
Score plot of the first three PCs considering the contribution of chemical and morphological parameters of particles sampled on the three investigated sites (a - differentiated by composition, b – differentiated by site of sampling).

Figures 10 and 11 (a - b), in which also the morphological parameters are considered in the PCA and explains 43% of the total variance, show that most of the particles belonging to the urban site differ from those of the other sites. This evidence is due mainly to the composition (they are often contaminated by metal elements of anthropic origin) and to the wider abundance of soot which is characterized by high loadings of morphological shape parameters.

## Experimental

Aerosol samples of PM10 were collected on 23 July 2008 on polycarbonate membranes (47 mm in diameter with 1 μm pore size, PALLFLEX Membrane Filters) and they were collected using a low volume sequential sampling system (sampler Hydra Dual Channel, air flow 2.3 m^3^/h). Filters were sampled for 24, 12 and 6 hours in order to evaluate the best condition for the single particles analysis. Morphological-chemical characterization of particles has been performed by a scanning electron microscope (SEM) FEI/Philips XL30 equipped with EDS microanalysis. 20 chemical parameters (C, O, Na, Mg, Al, Si, P, Cd, Cl, K, Ca, Sn, Ti, Cr, Mn, Fe, Co, Ni, Cu, Zn) were determined by SEM-EDS and 7 morphological parameters (Area, Aspect Ratio, Roundness, Fractal Dimension, Box Width, Box Height, Perimeter) were measured by Image Pro Analyzer 6.3 software. PCA and hierarchical and non-hierarchical CA were performed on data sets of each site using Statistica 6 software.

## Conclusions

In this work a protocol for the morphological chemical characterization of single particles has been developed. Different magnifications have been selected for SEM-EDS analysis to classify the size range of considered particles. Due to technical limits of SEM-EDS only particles with diameter more than 0.6 μm can be analyzed and this is the major limit of this work, because in this way we cannot study the finest particles which are of great interest in the study of particulate matter. In contrast SEM-EDS analysis, with the help of the multivariate statistical analysis of compositional and morphological data of particles, allows us to provide information about the sources of PM and to highlight differences and similitudes among sites. Moreover, morphological parameters allow us to discriminate among particles with the same chemical composition but with different morphology (see carbonaceous particles such as soot, cenosphere and biological one or spherical-irregular iron particles); this is a goal for the discrimination of pollution sources. Single particles studies, like this work, give useful information for the understanding of the formation of particulate matter but they are not exhaustive because they give information on a limited period of time and then they are complementary to other PM studies.

The investigated sites are mainly characterized by sea spray, crustal suspended matter, traffic related pollution and building activity.

In particular the crustal particulates of the rural and urban sites were mainly composed of Al, Si, K and in a smaller amount Al, Si, Ca probably these particles derive from the presence of minerals as KAlSi_3_O_8_ (orthoclase), KAlSi_2_O_6_ (leucite) and CaAl_2_Si_2_O (plagioclase); Fe and Mg were present in a minor amount and it was due to the presence of other types of crustal minerals such as (Ca_2_Mg_5_)Si_8_O_22_(OH)_2_ (amphibole), (Mg,Fe)_2_SiO_4_ (Olivine), (Mg, Fe)_2_SiO_3_ ( pyroxene) and K(Mg, Fe)_3_Si_3_O_10_(OH)_2_ (biotite). Some particles of SiO_2_ were also found. Particulates rich in calcium probably originated from CaCO_3_. In these two sites there were crustal particles deriving from local formations.

The yard site revealed a more heterogeneous situation and it was mainly due to the presence of a site characterized by earth-moving from local and non local areas, this is also due to the use of building materials characterized by a wide compositional range; in fact it was observed that, in addition to particles characterized only by Al-Si-K, there was a large presence of particles characterized by Al-Si-Ca and Si-O with the presence of elements such as Mg and Fe that characterize different kinds of crust.

Three kinds of carbonaceous particles were found: biological, soot and cenosphere. These particles were characterized by C and O, with minor amounts of other elements. Only the biological particles (e.g.: pollen) showed a slightly appreciable amount of Na, Ca, Cl, Mg and sometimes K. However, it is important to note that carbon soot particles were characterized by high values of Fractal Dimension and they were present in higher amounts in the urban site.

Metal particles were mainly characterized by iron; they were present in two different distinguished forms: spherical or angular particles. The spherical particles of iron were generated by iron fusion processes at high temperature (anthropic sources); while other kinds of iron metal particles can be linked: to a crustal origin, those enriched by typical terrigenous elements as Si, Al, K, and to an anthropogenic origin, as abrasion of metallic materials or in general to traffic related sources.

In addition, iron particles particulates composed of ZnCu were found and they were of an anthropogenic origin.

## Methods

SEM images have been obtained by a gaseous secondary electron detector (GSE) with an accelerating voltage of 30 kV. All the samples have directly been introduced in the microscope sample chamber without any further preparation. SEM Philips XL30 operates in the low vacuum (LV) mode (0.7 torr). In the LV mode, gas molecules surrounding the electron beam for specimen illumination are ionized and electrically neutralize charging of the specimen surface, thus, non conductive specimens can be observed without coating.

An X-Ray spectrum has been collected for 120sec for every SEM image. This allows us to recognize both the main elements and the trace elements. X-ray intensities were converted in wt% elements by a standarless ZAF quantification, as it is shown in literature for similar use of SEM analysis [18,29]; 20 chemical parameters (C, O, Na, Mg, Al, Si, P, Cd, Cl, K, Ca, Sn, Ti, Cr, Mn, Fe, Co, Ni, Cu, Zn) were determined by SEM-EDS and 7 morphological parameters (Area, Aspect Ratio, Roundness, Fractal Dimension, Box Width, Box Height, Perimeter) were measured by Image Pro Analyzer 6.3 software. For each particle, the calculated concentration for a specific element is quantified if it is larger than the standard deviation σ_b_ of the signal of the polycarbonate membrane. In cases in which the concentration is below the method detection limit (MLD), or not detectable above the average variability of the blank filter, a concentration value equal to the maximum between the MDL and σ_b_ has been assumed.

**Figure 12:**
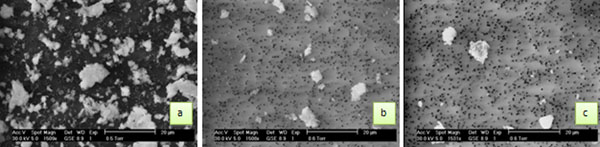
SEM images of PM10 filter (magnification 1500x) after (a) 24h of sampling, (b) 12h of sampling, (c) 6h of sampling.

In order to carry out the morphological and chemical analyses of the single particles, SEM images have to show particles well separated from each other and, moreover, the presence of particle clusters, which can be generated during long time sampling, have to be minimized. In order to choose the best sampling condition, filters sampled in each site for 24, 12 and 6 hours have been observed by SEM. SEM images of particles sampled in the yard site are shown in Figure 12. This site presents a higher amount of PM10 than the other sites and it is due to the construction activity in this site, so its SEM images of PM show a higher amount of particles and clusters than the other site images. 6 hours sampled filters were chosen because they present distinct particles and because the number of clusters is reduced in comparison to filters sampled for 12 and 24 hours. In this work filters sampled from 12:30 to 18:30 were analyzed. In order to study the atmospheric particles sampled in the same time period in the selected sites, 6 hours sampled filters of Antonelli Park (urban site) and St. Augustine (rural site) were also analyzed.

In order to obtain detailed SEM images for an accurate determination of morphological parameters, the image definition and the area of the particles are essential. Considering that in the scanning electron microscope the lateral resolution is often on the order of 100-200 nm for atmospheric aerosol particles with low contrast (i.e. particles from combustion processes) and that microanalysis of particles with diameter less than 0,6 µm gives signals not statistically distinguishable from background [30], then, five magnifications were determined and, for each one, particles with defined area and diameter were analyzed. The selected magnification of SEM images and the dimensions of the analyzed particles are: particles of area between 72.3 and 289.4 µm ^2^ (which correspond to 9.6-19.2 µm diameter respectively) have been observed with a magnification of 600x, particles area between 18.1 and 72.3 µm ^2^ (4.8 – 9.6 µm diameter) with a magnification of 1200x, particles area between 4.5 and 18.1 µm ^2^ (2.4 – 4.8 µm diameter) with a magnification of 2500x, particles area between 1.13 and 4.5 µm ^2^ (1.2 – 2.4 µm diameter) with a magnification of 5000x and then a particles area between 0.28 and 1.13 µm ^2^ (0.6 – 1.2 µm diameter) has been observed with a magnification of 10000x. These selected magnifications allow to analyze the chemical and morphological parameters of particles in the entire particle size range considered (with a continuous area and diameter range, 0.28 - 289.4 μm^2^ and 0.6 - 19.2 μm respectively).

About 80 particles were analyzed in each selected range and in each site. In total 495, 527 and 317 particles belonging to the yard, urban and rural sites, respectively, were analyzed.

PCA and hierarchical and non-hierarchical CA were performed on data sets of each site using Statistica 6 software. PCA analysis was performed on the concentration matrix following the data scaling procedure [31]. The elements that were not quantified (under MDL or comparable with signal of blank filter) in more than 20% of the samples have been excluded from the analysis similarly to the selection reported in literature [32]. For data not quantified values equal to the maximum between the detection limit and the standard deviation of blank filters have been used in the analysis [4]. The cluster analysis is an effective multivariate statistical method that could be used to achieve a greater confidence in the classification obtained with PCA. It has been shown that CA can assist PCA in the identification of clusters in atmospheric aerosol particles [33]. Therefore in this work the cluster analysis was essentially used for comparison purposes. Dendrogram has been obtained using Ward's method.

## Competing interests

The authors declare that they have no competing interests.

## Authors' contributions

FB, DA, carried out SEM-EDS measurements and in collaboration with MS, TS and MT elaborated images and data and drafted the manuscript. AG, CT, GM conceived the overall research and basing on their experience and relevant literature, contributed to the statistical analysis of data, interpretation and understanding of the experimental results and to the optimization and validation of the manuscript.

All authors have read and approved the final manuscript.
